# Editorial on “Elective Thoracic Surgical Resections for Pulmonary Arteriovenous Malformations—A 16 Year Single‐Center Experience”

**DOI:** 10.1002/pul2.70073

**Published:** 2025-04-02

**Authors:** Raghav Murthy

**Affiliations:** ^1^ Pediatric Cardiac Surgery, Medical City Children's Hospital Dallas Texas USA

The authors of this manuscript titled “Elective thoracic surgical resections for pulmonary arteriovenous malformations—a 16 year single‐center experience,” presents excellent surgical results for elective operations performed in 24 patients with pulmonary parenchymal arteriovenous malformations. The authors must be congratulated for their excellent results and follow‐up on these patients. The reader must be aware that the main manuscript is supplemented by an additional data supplement. The supplement reads as an entirely separate manuscript with a lot of additional details, including surgical techniques and outcomes. This is very unusual for a manuscript as the whole manuscript seems to contain two manuscripts within one. The reader needs to read the data supplement in depth to understand the nuances of the main manuscript.

The authors reviews 714 patients treated with pulmonary AVMs in their institution between 2006 and 2022. Intervention was undertaken in 555 patients while the remainder were managed conservatively. This manuscript details 24 patients who were managed with elective surgery out of those 555 patients. The authors used very selective criteria to determine what group of patients would benefit from surgery. All patients were discussed at the multidisciplinary team conference. The authors note that the patients who benefit from surgery are those in whom the right‐to‐left shunting cannot be completely abolished by embolization, but where limited surgical resection offers the possibility of a cure. Additional patients that might benefit from surgery are those who were not amenable to further embolization but have persistent symptoms, young women planning future pregnancies with hemoptysis or pulmonary AVM‐induced activity limitation, and to decrease radiation exposure in patients in the future. In the Results section, the authors note significant improvements in oxygen saturation between the preoperative and the postoperative groups, translating to improved exercise tolerance.

There are several important points I would like to make. This cohort of patients does not include pediatric patients. The cohort also does not include patients with congenital heart disease or patients with single ventricle palliation. It is very well‐known that the population of patients with congenital heart disease, especially those with single ventricle palliation, heterotaxy, status post Glenn operation, status post Fontan operation, have tendencies to develop pulmonary AVMs. This is especially true in heterotaxy patients where certain types of surgical palliation prevent the “hepatic factor” from reaching the lungs. “Hepatic factor” seems to prevent pulmonary AVMs and it is very well reported that when the “hepatic factor” is rerouted to the lungs, pulmonary AVMs decrease/disappear and oxygen saturation increases [1]. It is important to note that this manuscript does not reflect such patients.

There are known descriptions of pulmonary AVMs occurring in the trachea and bronchi. The current manuscript is limited to pulmonary parenchymal AVMs. The readers must note that there is an entity of AVMs that can occur intraluminally, within the airways, which could result in massive hemoptysis and can be lethal. A combination of interventional and surgical techniques can be used to manage these patients [2].

Overall, this is an extremely important manuscript that adds a lot of information to the existing literature. It clearly informs the reader of the patient that would benefit from surgical resection. It also highlights the importance of having a multidisciplinary team to obtain such excellent results. I am eager to read future manuscripts from this group of authors describing their series of emergency interventions in their cohort.

## Author Contributions

Raghav Murthy was the only author of this editorial.

## Ethics Statement

The author has nothing to report.

## Conflicts of Interest

The author declares no conflicts of interest.

## References

[1] T. Carberry , R. Murthy , A. Hsiao , et al., “Fontan Revision: Presurgical Planning Using Four‐Dimensional (4D) Flow and Three‐Dimensional (3D) Printing,” World Journal for Pediatric & Congenital Heart Surgery 10, no. 2 (March 2019): 245–249, https://pubmed.ncbi.nlm.nih.gov/30630383/. 30630383 10.1177/2150135118799641

[2] A. Sengupta , O. Aljohani , H. El‐Said , A. Rao , M. Brigger , and R. Murthy , “Sleeve Lobectomy for an Arteriovenous Malformation in the Bronchus Intermedius in a Child,” Journal of Pediatric Surgery Case Reports 45 (June 2019): 101207.

